# Great Men Great Sufferers

**Published:** 1864-12

**Authors:** 


HALL'S JOURNAL OF HEALTH.

VOL. XI.] DECEMBER, 1864. [No. 12.

### GREAT MEN GREAT SUFFERERS.

"Killed with hard study," is the verdict often rendered as to many young men, who die while pursuing their studies, or soon after entering their profession ; the same is said of many literary men. Multitudes of clergymen, who are never well, have the reputation of having been brought into this condition by overwork, by having to study so hard. Many a man of a great mind and of eminent usefulness is a martyr to some form of human suffering, to some malady, which, in the slow progress of years, is permanently undermining the constitution and eating into the very vitals.

Great business men, who have large interests on their minds all the time, are sometimes sent by writers to the insane asylum, as in a subsequent article in this number on "Health, Happiness and Gold," as a result of excessive mental application to one great absorbing idea. It is a rare thing to find any man eminent for his talents, who is not either a drunkard practically, or a sufferer from some physical malady. Hugh Miller was the victim of dyspeptia ; the tortures of which, finally crazed his brain, and led him to suicide. The most eloquent man that ever lived in any age or nation, on astronomical subjects, and whose indomitable energy and tenacity of purpose, are illustrated in some of the pages following, as an encouragement to those of our young readers who are ambitious that the world should be benefited by their having lived in it, was for many of the last years of his life the helpless sufferer from dispeptic horrors, enough to drive any mind mad, less great than his.

Great students have great appetites. Hard study makes a healthy man as hungry as hard work. The employment of the brain, hard thinking, exhausts a man's strength as certainly and completely as any form of bodily labor. Girls and boys and young men at school, academy, or college, are always ready to eat something, and can eat almost anything, for a while after they have entered upon their studies.

Whether a man kills himself with whiskey, or tobacco, or food, the crime is the same. A man is equally a suicide, whether by drinking, smoking or gluttony. Excessive smoking, excessive drinking, excessive eating, are the results of an abandonment to an animal appetite, to an animal indulgence, such indulgence is beastly, it is ignoble, it is pitiful. Pitiful and sorrowing is it to contemplate the spectacle of a magnificent mind going out in premature darkness, in consequence of its low indulgence of the animal propensities. Our eminent men in law, physic and divinity, in finance and politics, and on the editorial chair, when they are disabled or die prematurely, or pass their last days behind the iron bars of an asylum, are the victims, in almost all cases, not of hard study but of hard drinking, eating or other form of over indulgence, whether in opium, spirits or tobacco, and the disgraceful fact ought to be more generally understood than it is. The process is as follows. Study is a fascination. It has been said of the great Newton, that he had often to be reminded that it was the dinner hour. Writers of prose, poetry and music are conscious that many times the call to dinner has been an unwelcome one, and that if they had been unusually interested in the investigation of a subject, the mind leaves it most reluctantly, if indeed at all ; food is eaten without any perceived taste, is swallowed without satisfaction, and no sooner is the "process" of dining over, than the student hastens to his writing or his book, to pursue the chase of ideas, and revels in these, with all the abandon of his nature; the result is, that the nervous energy which ought to have gone to the stomach to be expended in withdrawing nutriment from the food, and supplying a pure blood to feed the system and repair its wastes, is expended on the brain ; the food is thus allowed to remain in the stomach insufficiently acted on, is imperfectly digested ; the stomach robbed of its due proportion of nervous energy, endeavors to get through its work, but for want of power, of strength, does it imperfectly ; all the work is done after a fashion, but none of it is well done; none of the materials for making good blood are perfect materials, hence the blood itself is imperfect, is impure, is thick ; does not flow through the channels of the system with life like activity ; but passes along slowly, sluggishly ; and in process of time, congests, dams up, scarcely flows at all, and finally stagnates, ceases to flow altogether, and death ends the history. But it is curious to know that the blood thus stagnates in different parts of the system, in different persons, according as some one part of such system has been rendered weak, either from hereditary influences, or by accidental circumstances.

If the lungs have been rendered the weakest part from too close confinement to the study and a want of active out-door exercise, the stagnation induces hemmorrhage ; coming upon a man sometimes with the surprise of a clap of thunder under a clear sky.

If the brain has been rendered weak by its share of nervous energy having been sent habitually to an overloaded stomach, or to over-worked muscles, the blood dams up there, and there is paralysis or appoplexy ; which is quite a common ending to late dinners, and to those who feed on oysters, lobsters and turtle soup at eleven o'clock at night.

If the throat has been rendered weak by the injudicious or excessive use of the voice, and going out into the cold air, or riding against the wind, before the parts have cooled and rested, the congestion begins there, causing smarting, burning, pricking or some kind of clogging up of the throat, or voice organs, to end in a permanent disablement of the voice, in an ulceration of the parts and death by starvation, from an inability to swallow food ; or, passing down to the lungs, fell consumption winds up the sad history.

If the lower bowels have become weakened by sitting habitually on warm cushions ; or by having allowed habits of constipation (that is want of a daily action of the bowels) to grow upon the system, the congestion begins there, inducing piles ; or the more perilous fistula ; which, if uncured by the surgeon's knife, at an expense of several hundred dollars, induces the deplorable condition, where the urine and excrements come away by driblets all the time ; and the grown man must be "diapered" for the remainder of his life.

Students and sedentary persons become the victims of constipation, in nearly all cases as follows : When interested in a composition or an investigation or having an appointment, nature may give one of her calls, but the urgency of the matter in hand induces the deferment of that call for five minutes, or half an hour or longer ; nature thus baffled, ceases to urge again, for several hours ; her habit of regularity has been broken into ; the next day her call is later, and later ; finally passes to the second or third day ; sometimes only once or twice a week, and the health is ruined forever ; thus, if nature is not instantly obeyed when her instinct indicates that something should be passed out of the system as an incumbrance, as rubbish in a house, or workshop, she begins to dispose of it in another direction, by setting up a heat in the parts ; this heat causes the more watery particles of the matter, above referred to, to be reabsorbed and sent to the blood, to render it more thick, and impure, and to increase the original malady ; to add fuel to the flame. The blood is not only rendered impure, but part of the matter which ought to have gone down into the privy, has been circulated through the system again and goes to the mouth and lips and tongue ; goes all over the body ; no wonder that habitually constipated persons have ailments all over the body, have an "odor" about them, which heralds their approach when a mile away, more or less ! Therefore, the great man owes his sufferings more to hard eating, than to hard study ; more to the filthy habit of postponing nature's calls, than to over-work; and hence merits our contempt, more than our sympathy.

